# The role of nasal and gut microbiota in allergic rhinitis: immunomodulation and therapeutic prospects

**DOI:** 10.3389/fimmu.2026.1984960

**Published:** 2026-10-30

**Authors:** Yali Yan, Liang Bao, Xiao Chen, Huiying Xiao

**Affiliations:** 1 Inner Mongolia Key Laboratory of Allergic Diseases, Hohhot First Hospital, Hohhot, China; 2 Medical Innovation Center for Nationalities, Inner Mongolia Medical University, Hohhot, China; 3 Ministry of Scientific Research, Hohhot First Hospital, Hohhot, China

**Keywords:** allergic rhinitis, dysbiosis, early-life exposure, immunomodulation, microbiota, microbiota-targeted therapy

## Abstract

Allergic rhinitis (AR) is a prevalent immunoglobulin E (IgE)-mediated inflammatory disorder of the nasal mucosa that imposes substantial global health and socioeconomic burdens. The pathogenesis of AR involves complex interactions among epithelial barrier dysfunction, immune dysregulation, and environmental factors. Emerging evidence underscores the critical role of the human microbiota, particularly communities residing in the nasal cavity and gut, in modulating immune homeostasis and susceptibility to AR. Early-life microbial exposure profoundly shapes initial microbiota colonization. Observational and longitudinal studies associate nasal and gut dysbiosis with impaired immune tolerance and an increased risk of AR, although the causal direction of these associations remains incompletely established. At a mechanistic level, microbial communities may influence AR through short-chain fatty acids and other metabolites, epithelial barrier integrity, and an integrated innate/adaptive network comprising type 2 innate lymphoid cells, dendritic cell subsets, Th 1, Th2, Th17, and regulatory T cells, together with epigenetic regulation. Among microbiota-targeted interventions, selected probiotic strains and bacterial lysates show modest, strain- and regimen-specific benefits in some randomized trials, whereas fecal microbiota transplantation remains experimental in AR, with current support extrapolated from other conditions and preclinical models. This review synthesizes current insights into microbiota-AR interactions and their underlying mechanisms, explicitly distinguishing human, preclinical, and mechanistic evidence, and critically appraises the therapeutic potential and limitations of microbiota-targeted strategies for AR prevention and treatment.

## 1 Introduction

Allergic rhinitis (AR) is an immunoglobulin E (IgE)-mediated inflammatory response of the nasal mucosa caused by allergen exposure. Globally, AR affects approximately 10%-30% of adults and up to 40% of children, with epidemiological research indicating an increasing prevalence (1, 2). These pooled estimates conceal substantial geographic, age-related, and diagnostic heterogeneity: prevalence differs across world regions and between urban and rural settings, and between survey-based and sensitization-based case definitions; children and adolescents are generally more affected than older adults, and time trends vary by region. AR symptoms significantly impair multiple health domains, including quality of life, sleep, affective state, cognitive performance, and sexual function, and are frequently associated with asthma, allergic conjunctivitis, and sinusitis, resulting in significant healthcare burdens and socioeconomic consequences (3–5). Current management strategies primarily include pharmacotherapy, environmental control, and allergen immunotherapy (6). Although these treatments can mitigate symptoms, they are often limited by adverse effects and incomplete disease control.

Due to the limitations of current therapies, there is increasing interest in alternative AR strategies. Among these, the human microbiome, particularly in the gut and nasal cavities, is a primary focus, as it critically regulates immune homeostasis. Dysregulation of this microbial ecosystem may contribute to the pathogenesis of allergic disorders. We critically synthesize the association between the microbiome and AR, separating human, preclinical, and mechanistic levels of evidence, to elucidate underlying pathways and assess the therapeutic potential of microbiota-targeted interventions. Finally, we seek to translate microbiome discoveries into new preventive approaches and targeted therapies for AR, potentially improving clinical outcomes and patient-centered metrics (7).

## 2 Literature search strategy

We searched PubMed/MEDLINE and Web of Science from inception through August 2026 using combinations of the terms “allergic rhinitis” OR “hay fever” OR rhinitis, cross-combined with “microbiota” OR “microbiome” OR dysbiosis OR probiotic OR “bacterial lysate” OR “fecal microbiota transplantation” OR “short-chain fatty acid”. The reference lists of retrieved articles and of relevant systematic reviews and meta-analyses were hand-searched to identify additional studies.

Only peer-reviewed English-language publications were considered, and conference abstracts without accessible full data were excluded. Human studies, animal models, and mechanistic or *in vitro* work were retained but tagged by evidence type and are presented separately. Priority was given to systematic reviews and meta-analyses, randomized controlled trials (RCTs), large or longitudinal cohorts, and mechanistically seminal studies; small case series and single-case reports were used only to illustrate hypotheses.

## 3 Etiology and pathogenesis of AR

Nasal epithelial cells provide the first line of immunological defense against inhaled allergens, and they play an important role in AR pathogenesis. These specialized cells are strategically located in the nasal mucosa and interact directly with diverse environmental allergens. Allergens deposited in the respiratory system initiate a sequential immune cascade that involves both innate and adaptive immunity. This immunologic activation is a fundamental mechanism of the allergic response and a key factor in AR development, emphasizing the critical role of nasal epithelial cells in respiratory allergies.

AR pathogenesis primarily involves distinct T helper (Th) cell subsets and their unique cytokine profiles. Allergens cause epithelial cells to release alarmins such as interleukin (IL)-33, IL-25, and thymic stromal lymphopoietin. These alarmins activate type 2 innate lymphoid cells (ILC2s) to secrete canonical Th2 cytokines, including IL-13 and IL-5. Antigen-presenting cells (APCs), especially dendritic cells (DCs), are activated simultaneously and play a pivotal role (8). AR is therefore not adequately described as a simple Th1/Th2 imbalance: it is a networked type 2 response in which DCs subsets, regulatory T cells (Tregs), Th17 cells, epithelial alarmins, ILC2s, neuroimmune crosstalk, and epithelial barrier integrity jointly shape disease expression and severity. Activated DCs participate in immune surveillance by recognizing and phagocytosing allergens. Following antigen processing, DCs present peptide-major histocompatibility complex class II complexes to naive CD4+ T cells, causing them to activate and differentiate (9, 10). IL-4 induces Th0 differentiation into Th2 by activating GATA-3 and STAT6 signaling pathways (11). This differentiation produces characteristic type 2 cytokines, such as IL-4, IL-5, and IL-13. In parallel, tolerogenic DCs, together with retinoic acid and microbial metabolites such as short-chain fatty acids (SCFA), promote differentiation of induced Tregs that normally constrain type 2 responses; deficient or unstable Treg control is increasingly implicated in AR. IL-5 specifically mediates eosinophil recruitment, promoting their bone marrow production, maturation, and inflammatory tissue migration, emphasizing its role in allergic response. Additionally, IL-4 and IL-13 promote B cell proliferation and plasma cell differentiation, enabling allergen-specific IgE (sIgE) production (12). These IgE antibodies circulate systemically, binding FcϵRI receptors on tissue-resident basophils and mast cells (MCs) (13). This sensitization prepares these cells for stronger responses to allergen re-exposure (**Figure 1**).

**Figure 1:**
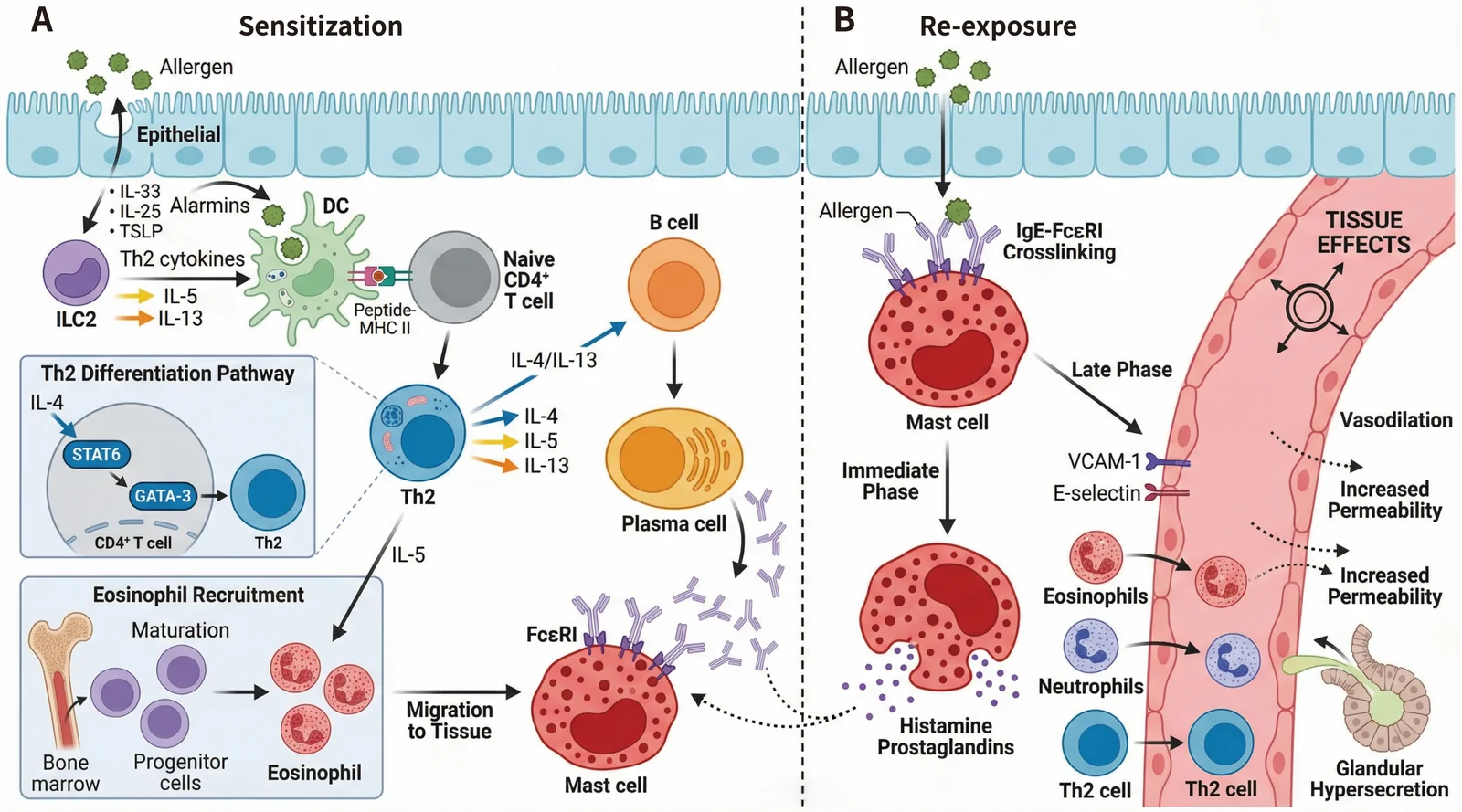
This figure illustrates the two core phases of AR: the sensitization phase **(A)** and the re-exposure effector phase **(B)**. **(A)** Sensitization: Allergen translocation across the epithelial barrier induces a type 2 immune response, resulting in allergen-sIgE synthesis, MC sensitization, and eosinophil mobilization. **(B)** Re-exposure: Cross-linking of IgE-FcϵRI complexes on MCs by the allergen triggers degranulation, driving immediate tissue responses and late-phase inflammatory cell recruitment. Abbreviations: AR, allergic rhinitis; IgE, immunoglobulin E; sIgE, specific immunoglobulin E; MCs, mast cells; Th2, T-helper 2 cell; IL, interleukin.

Following re-exposure to the same allergen, the IgE-FcϵRI complex on MCs or basophils cross-links, causing cellular activation and degranulation. This results in the release of preformed mediators such as histamine, prostaglandins, leukotrienes, bradykinin, and other inflammatory mediators. This early-phase response appears clinically as rhinorrhea, sneezing, and nasal pruritus within minutes (14). During the late-phase response, these mediators induce vascular endothelial cells to upregulate the expression of vascular cell adhesion molecules and E-selectin. This promotes leukocyte rolling and firm adhesion to the endothelium. Chemoattracted by cytokines such as IL-5, these leukocytes—predominantly eosinophils, neutrophils, and Th2—transmigrate into the nasal submucosa, causing chronic inflammation. Their subsequent mediator release causes vasodilation, enhanced vascular permeability, and hypersecretion, resulting in nasal obstruction (15).

AR is a chronic upper airway inflammatory disorder involving multiple immune cells and cytokine networks (16). The increasing global prevalence of allergic diseases is due to complex gene-environment interactions, with epigenetic regulation of susceptibility genes playing a critical role (17). Recent genomic advances have identified 446 AR-associated genes through protein interaction network analysis using random walk algorithms. IRAK2 and MAPK signaling pathways are important pathogenic mediators, which critically regulate allergic inflammatory responses in AR (18). In Chinese cohorts, IL-33 gene polymorphisms and haplotypes demonstrate significant AR risk associations, emphasizing its importance in genetic predisposition (19).

Epigenetic mechanisms, including microRNAs (miRNAs), link environmental exposures to AR-relevant gene expression; however, most reported miRNA changes come from cross-sectional or animal data and cannot yet be distinguished as causal drivers from compensatory or downstream responses. MiR-29a-3p maintains nasal epithelial barrier function by targeting CTNNB1-VCL complexes, which regulate paracellular permeability (20). Bioinformatic analyses by Liu revealed that miR-17-5p may impact seasonal allergic rhinitis by targeting ABCA1 and CD69 (21). MiR-223-3p increases allergic inflammation through FBXW7-mediated enhancement of eosinophil degranulation (22). Exosomes derived from T follicular helper cells impair DCs maturation via the miR-142-5p/CDK5/STAT3 axis, a pathway critically involved in AR pathogenesis (23). MiR-146a reduces AR symptoms through TLR4/TRAF6/NF-κB inhibition, reducing both ovalbumin (OVA)-sIgE levels and inflammatory cytokines in MCs and basophils (24). MiR-16 inhibits IκB kinase, suppressing IL-13-induced NF-κB activation and subsequent production of key inflammatory mediators, including *GM-CSF*, *eotaxin*, and *MUC5AC* (25).

Environmental determinants include both indoor and outdoor allergens, air pollutants, climate change, and microbial exposure, all of which have a significant impact on immune development and clinical manifestations from prenatal stages to early childhood (26). Compelling evidence confirms environmental exposures as key drivers of pathogenesis of allergic diseases, with childhood serving as a critical window of vulnerability (27). Airborne allergens, ambient pollutants, and meteorological factors are the primary etiological factors in AR development (28). Inhaled aeroallergens, such as mold spores, animal dander, and pollen grains, are primary AR triggers through mucosal immunomodulation (**Figure 2**). An epidemiological study of 40,279 Chinese adults identified household moisture, indoor mold colonization, and window condensation as potential AR risk factors (29). Emerging evidence indicates childhood exposure to animal dander may provide immunoprotective effects against AR development (30, 31). Pollen allergen profiles demonstrate significant geographical heterogeneity, with region-specific dominant species: birch and grass pollen dominance in Denmark, Japanese cedar pollen in Japan, and Artemisia annua predominance in northern China (32–34). Despite their theoretical efficacy, the practical implementation of environmental allergen mitigation strategies remains operationally challenging (35).

**Figure 2:**
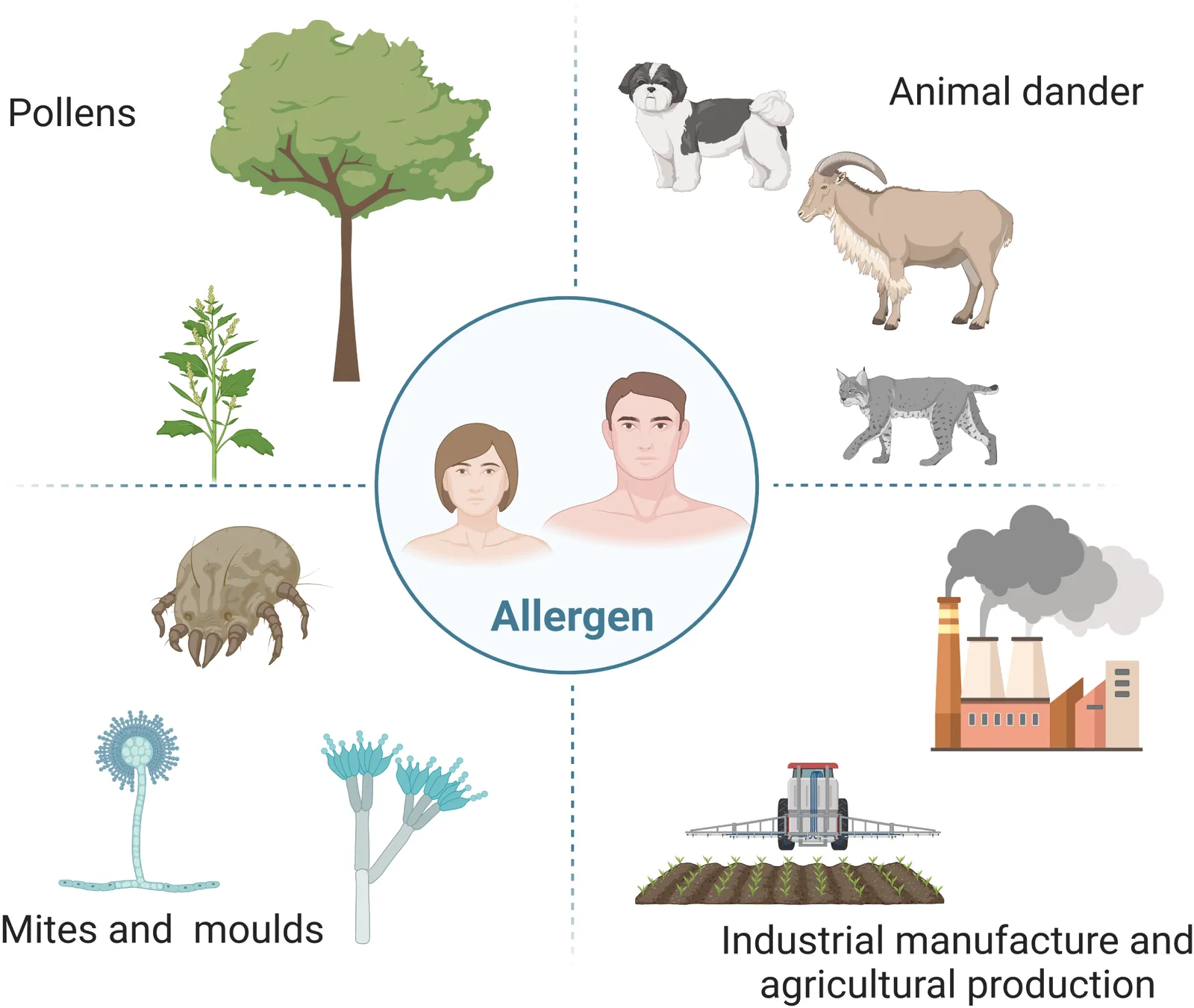
Major categories and sources of common environmental allergens. AR is predominantly triggered by environmental allergens, including pollen grains, dust mite feces, fungal spores, animal-derived proteins, and anthropogenic air pollutants. These allergens provoke type 2 inflammatory responses in the nasal mucosa through IgE-mediated hypersensitivity, clinically presenting with paroxysmal sneezing, watery rhinorrhea, nasal obstruction, and pruritic symptoms. AR, allergic rhinitis; IgE, immunoglobulin E.

Elevated ambient air pollutant concentrations correlate significantly with the increased prevalence of allergic respiratory diseases (36). Pediatric studies have confirmed that ozone exposure is an independent risk factor for allergic sensitization (37). Ozone and particulate matter demonstrate significant dose-response relationships with the incidence of allergic conjunctivitis (38). A bidirectional relationship exists between atmospheric pollution and climate change, with significant clinical implications. Primary air pollutants influence climate systems, whereas climatic changes modify allergen phenology by extending pollen seasons, intensifying allergenicity, and expanding geographic distributions (39, 40). Climate change has increased birch pollen concentrations and prolonged exposure durations, thereby elevating sensitization rates (41). Meteorological parameters, including temperature, precipitation, wind patterns, and barometric pressure in northern China’s grasslands, modulate pollen exposure dynamics, directly impacting AR epidemiology (42). Temperature increases were significantly positively associated with the incidence of allergic respiratory diseases. A meta-analysis demonstrated a 29% increased risk of allergic respiratory outcomes per 1 °C increase in temperature (43). Crowdsourced symptom data have revealed that temperature and wind speed correlate strongly with indices of allergic symptom severity (44). Thus, environmental determinants affect both the development and clinical severity of AR. Environmental exposures may drive disease progression through allergen-specific epigenetic modifications (45). N95 mask use significantly reduced AR symptoms in healthcare workers, without comparable ocular symptom relief (46). Climatic variability shapes the nasal microbiome composition, potentially modulating the pathogenesis of allergic respiratory diseases. Geographically, specific insights could enable precision medicine approaches for AR management.

In summary, the pathogenesis of AR involves a complex network of immune cells and signaling pathways. Recently, increasing evidence has indicated that the nasal and gut microbiota serve as critical interfaces between environmental factors and the host immune system, playing a central role in AR pathogenesis by modulating immune balance and inflammatory pathways. The following sections elaborate on the role of microbiota in AR.

## 4 The role of microorganisms in AR

### 4.1 The early-life microbiota

Recent research has moved beyond the traditional IgE-mediated allergic reaction mechanisms to investigate the critical role of the microbiome in the pathogenesis, diagnosis, and treatment of AR (47). Over the last three decades, genetic risk factors for allergic diseases in infants and young children have remained relatively constant. Environmental and dietary changes have increased the prevalence of allergic diseases while also having a significant impact on the gut microbiota (38, 48). The gut microbiota is a critical determinant of health, influencing metabolic and inflammatory responses and playing a key role in modulating intestinal barrier function (49). Numerous studies have found associations between the microbiome and various exposure factors, such as delivery mode, feeding practices, probiotic or prebiotic administration, antibiotic and other medication use during pregnancy and infancy, and early-life environmental conditions (**Table 1**).

**Table 1:** Selected early-life exposures associated with microbiota development and allergic outcomes.

Exposure factor	Microbiota	Population/timing	Study design	Key findings	Ref.
Cesarean section	Oral bacteria	Newborn	Cross-sectional comparison	*Petrimonas*↑, *Bifidobacterium*↑, *Desulfovibrio*↑, *Pseudomonas*↑, *Staphylococcus*↑, *Tepidmicrobium*↑, *VadinCA02*↑, *Bacteroides*↑	(110)
Probiotics	Gut microbiota	At 5 days, 10 days, and 30 days of age	Longitudinal, preterm infants	*Weissella*↓, *Veillonella spp*↓, *Klebsiella*↓	(111)
Antibiotics/Human milk	Gut microbiota	At 1 month, 6 months, 12 months, and 24 months of age	Longitudinal birth cohort	*Lachnospiraceae (unclassified)*↓, *Ruminococcaceae*↓, *Firmicutes*↓, *Fusobacterium*↑, *Lentisphaerae*↑	(112)
Per- and polyfluoroalkyl substances	Gut microbiota	At 6 weeks and 1 year old	Longitudinal birth cohort	*Bacteroides vulgatus* ↓	(113)
Antibiotics	Nasal microbiota	At 2 months, 13 months, and 24 months of age	Population-based longitudinal cohort	*Haemophilus*↑	(114)
Furry pets	Meconium microbiome	Newborn	Population-based cohort	*Bacteroidetes*↑	(115)
Furry pets	Gut microbiota	At age 3 to 4 months	Birth cohort	Ruminococcus↑, Oscillospira↑	(116)
Vitamin D	Gut microbiota	At age 3 to 6 months	Birth cohort	*Lachnospiraceae/U. Clostridales* ↑, *Lachnobacterium* ↑, *Lactococcus* ↓	(117)

The first 1,000 days of life are critical for establishing the gut microbiota and the development of immune tolerance (50). Alterations in the composition of early-life intestinal microbiota can cause intestinal microecological imbalance, disrupting the regulatory systems that maintain Th1/Th2 equilibrium and potentially increasing allergic susceptibility (51). Studies have revealed that the abundance of specific bacterial genera in children exhibits long-term changes from infancy to preschool age, implying a strong association between early gut microbiota composition and subsequent allergic development (52, 53). This association suggests that early microbial exposure may affect immune responses and allergic risk. Furthermore, the microbiome in infants and young children is generally less diverse and stable than in adults. The association between gut microbiota composition and the development of allergic diseases or asthma is stronger in neonates than in older children, implying that early exposure to specific allergens and bacteria may provide protective effects (54).

Environmental and/or genetic factors that disrupt intestinal function can affect Th1/Th2 balance, resulting in impaired immune tolerance, disrupted normal allergen clearance, and increased AR susceptibility (55). Studies indicate that prenatal PM2.5 exposure increases the risk of AR in preschool children. However, extended exclusive breastfeeding may reduce the incidence of allergic disorders in children exposed to air pollutants (56).

The dynamics of the oral and gut microbiome during early childhood have a significant impact on immune maturation processes and allergic predisposition (57, 58). A 13-year Finnish prospective probiotic-intervention cohort followed children from infancy into adolescence and reported persistent associations between infant gut microbiome profiles and the incidence of AR. Affected children had reduced populations of *Bifidobacterium* and *Escherichia/Shigella*, and increased *Bacteroides* populations (59). The hygiene hypothesis holds that microbial exposure protects against the development of allergies and asthma, with perinatal and/or early-life microbial exposure affecting physiological development. Early-life exposure to agricultural environments or high microbial/allergen loads may reduce the risk of childhood asthma and other allergic disorders (60).

Epigenetic modifications further connect early-life microbiota to AR. In the Copenhagen Prospective Studies on Asthma in Childhood birth cohort, the upper airway microbiota was sampled longitudinally at 1 week, 1 month, and 3 months of age and related to AR diagnosed by age 6. Findings indicated that lower microbial diversity at 1 week was associated with AR by age 6, which was partially mediated by altered DNA methylation patterns in upper airway mucosal cells (61). As an observational longitudinal association, however, methylation could itself reflect unmeasured environmental confounders. Zhang used the methyl-target method to analyze Chinese children aged 3 to 6 years with AR and discovered that early pet exposure and low ADAM33 methylation levels were associated with AR risk (62). These findings suggest that early-life microbiota may exert long-term effects on AR onset through epigenetic mechanisms, emphasizing the importance of microbial exposure in allergy prevention. Natural vaginal delivery, breastfeeding, and other interventions can help establish a healthy microbial community, which promotes normal immune system development (**Figure 3**).

**Figure 3:**
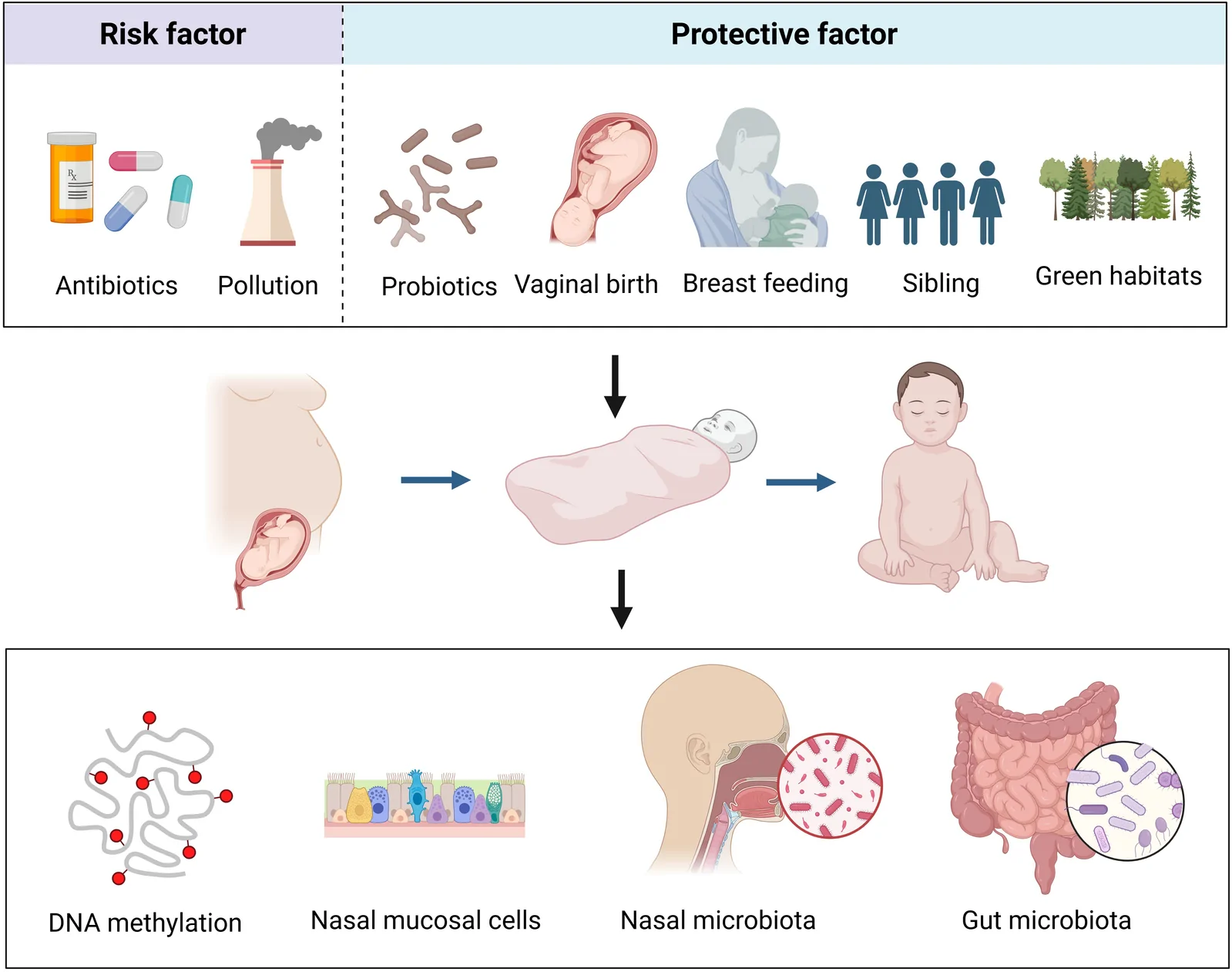
Early-life environmental factors shape mucosal microbiota and host developmental features. Early-life risk factors (antibiotics, pollution) and protective factors (probiotics, vaginal birth, breastfeeding, siblings, green habitats) modulate host developmental trajectories from gestation to childhood, and further impact DNA methylation, nasal mucosal cells, as well as nasal and gut microbiota. AR, allergic rhinitis.

### 4.2 Environmental microorganisms

Microbial diversity is associated with the incidence of AR. In a pooled analysis of two European birth cohorts (the Finnish rural-suburban LUKAS cohort, n = 312, and the German urban LISA cohort from Munich and Leipzig, n = 248), higher bacterial richness in indoor floor dust collected when children were 2–3 months old was associated with a lower risk of AR by age 10 in multivariable logistic models (in LISA, adjusted OR 0.12, 95% CI 0.05-0.29 for the highest vs lowest tertile, and 0.19, 95% CI 0.09-0.42 for the middle tertile), with a concordant trend in LUKAS (63). A cross-sectional study of 347 junior high school students in Malaysia identified associations between environmental microbiota and AR and non-allergic rhinitis (NAR). *Gammaproteobacteria* abundance was negatively correlated with AR and NAR symptoms, whereas higher overall fungal richness was positively associated with these symptoms. *Brasilonema bromeliae* and *Aeromonas enteropelogenes* were negatively, and *Deinococcus* positively, associated with AR and NAR (64). As a cross-sectional study, however, it cannot establish temporal order. These findings highlight the complex relationships among AR, NAR, and microbiota that could inform mechanistic understanding and targeted treatments.

### 4.3 Nasal microorganisms

The microbiome exhibits greater genetic diversity than the human genome and is essential for the development, function, and regulation of the immune system (65). Dysregulated interaction between the microbiota and the immune system under specific environmental contexts is thought to contribute to the pathogenesis of allergic diseases in individuals with genetic predisposition (66). Converging but heterogeneous studies report that dysbiosis across body sites, including the nasal cavity, intestines, lungs, and skin, is associated with, and sometimes precedes, allergic sensitization and inflammation (67–69). A recent study found that the composition and function of the upper airway microbiome may influence asthma development in both young and older adults (70).

Current research suggests an association between nasal microbiota dysbiosis and AR, emphasizing the microbiota’s role in modulating local immune responses within the nasal cavity. Most, although not all, cross-sectional studies report differences in nasal microbiota composition between healthy individuals and patients with AR, but the reported taxa and directions are not fully consistent across cohorts. The composition and diversity of these microorganisms may affect immune responses and inflammation in the nasal cavity, exacerbating allergic symptoms (71). At the phylum level, the relative abundance of *Actinobacteria*, *Bacteroidetes*, *Firmicutes*, and *Proteobacteria* in the nasal passages of patients with AR changes significantly (72, 73). At the genus and species levels, the abundance of pathogenic bacteria such as *Staphylococcus aureus*, *Moraxella catarrhalis*, and *Streptococcus salivarius* increases significantly, while protective symbiotic bacteria, including *Corynebacterium* and *Dolosigranulum pigrum*, decrease (74, 75).

Chiang observed that patients with AR had increased abundances of *Acetobacter*, *Mycoplasma*, *Escherichia*, and *Halomonas*, whereas healthy controls had higher abundances of *Zoogloea*, *Streptococcus*, *Burkholderia*, and *Pseudomonas* (76). Similarly, a study analyzing nasal mucus samples from 42 patients with AR and 30 healthy controls found that the *Firmicutes* phylum was significantly more prevalent in patients with AR, whereas *Proteobacteria* and *Actinobacteria* dominated the healthy cohort (77). Miao identified a correlation between reduced nasal microbial diversity and increased abundance of *Streptococcus salivarius*, a commensal bacterium linked to AR development, inflammatory responses, and nasal epithelial remodeling (75). Other studies reported higher abundances of *Vibrio vulnificus* and *Acinetobacter baumannii* in the nasal cavities of patients with AR, alongside lower abundances of *Lactobacillus murinus*, *Lactobacillus iners*, *Proteobacteria*, *Pseudomonadales*, and *Escherichia coli* (78). Teng discovered that the average relative abundance of *Ralstonia* in the moderate-to-severe AR group was higher than in the mild AR group, and that its abundance was positively correlated with the total nasal symptom score (79). Taken together, these findings suggest that variations in the nasal microbiota may be relevant to AR.

Importantly, no clinically validated taxonomic signature of AR currently exists. Nasal microbiome findings are highly sensitive to the sampling site, swab technique, sequencing platform and targeted 16S ribosomal RNA (16S rRNA) region, sequencing depth, age, season, geography, allergen exposure, intranasal corticosteroid or antihistamine use, intercurrent infection, and AR phenotype and severity. **Table 2** summarizes the principal human nasal studies and illustrates how differences in design contribute to apparently divergent results; these studies are predominantly cross-sectional, rely on relative abundance, and vary widely in sample size, which precludes pooling into a universal AR signature.

**Table 2:** Principal human nasal microbiome studies in AR.

Country; design	Participants; age	Sampling site & method	Sequencing approach	Principal findings	Ref.
USA; cross-sectional	11 AR, 8 healthy, 46 CRS; adults	Endoscopically guided top/inferior meatus swabs	16S rRNA, V3-V4	*Corynebacterium* dominates in healthy subjects, *Staphylococcus* is enriched in AR, and *Pseudomonas* and *Anaerococcu*s show higher abundance in CRS patients.	(71)
China; cross-sectional	32 AR, 20 controls; adults	Nasal secretions	16S rRNA plus untargeted metabolomics	Altered microbiota-metabolite interactions; β-diversity shift without consistent α-diversity difference.	(73)
Human cohort	55 AR, 105 healthy; adults	Nasal swabs	16S rRNA	*S. salivarius* associated with AR and shown to promote inflammation and nasal epithelial remodeling.	(75)
Taiwan, China; cross-sectional	20 AR, 19 healthy adults	Nasal extracellular vesicles	16S rRNA profiling of EVs	AR patients exhibit lower alpha diversity and altered microbial profiles in nasal EVs, with enrichment of *Acetobacter*, *Mycoplasma*, *Escherichia*, *Halomonas*, and depletion of *Zoogloea*, *Streptococcus*, *Burkholderia*, and *Pseudomonas*, alongside differential microbial metabolic pathways.	(76)
Korea; cross-sectional	42 AR, 30 healthy; adults	Nasal mucus	16S rRNA.	*Firmicutes* were more prevalent in AR patients, while *Proteobacteria* and *Actinobacteria* were dominant in controls, and *Staphylococcus aureus* characterized nasal dysbiosis.	(77)
China; cross-sectional	35 AR, 35 NAR, 20 healthy; adults	Nasal secretions of lower turbinate area	16SrDNA and metagenomic sequencing	Nasal microbiota profiles differ significantly between AR and NAR; *Staphylococcus* increases and *Corynebacterium* decreases in AR, differential taxa correlate with serum IgE, and nasal microbiota-based model distinguishes the two rhinitis groups.	(78)
China; cross-sectional severity study	45 AR children, 40 controls; children	Nasal swabs	16S rRNA	*Ralstonia* abundance rose with severity and correlated with TNSS.	(79)

A further interpretive caveat is reverse causality. AR itself remodels the nasal environment through inflammation, mucus hypersecretion, epithelial barrier disruption, sneezing, and rhinorrhea, and altered local nutrient availability. Moreover, intranasal corticosteroids, oral and topical antihistamines, antibiotics, saline irrigation, and allergen immunotherapy can themselves reshape microbial communities. Nasal dysbiosis observed in AR may therefore be partly a consequence of disease or therapy rather than its cause; longitudinal pre-treatment sampling and treatment-stratified analyses are required to disentangle these possibilities.

### 4.4 Gut microorganisms

A vast consortium of microorganisms colonizes the human gastrointestinal tract, with estimates of up to 100 trillion microbial cells. This region is particularly rich in immune cells, accounting for approximately 60%-70% of the body’s mucosal immune compartment. The microbiota influences various components of the immune system both directly and indirectly. Intestinal dysbiosis can perturb the Th1/Th2/Treg balance during immune development, reducing immune tolerance, impairing allergen clearance, and increasing susceptibility to AR (80). The gut microbiome plays an important role in modulating mucosal immunity and has been associated with AR mechanisms (81, 82).

A frequently cited summary index is the *Firmicutes* to *Bacteroidetes* ratio, but this ratio is not a validated biomarker of dysbiosis: it varies widely with age, diet, body mass, geography, and disease state and should not be treated as a definitive indicator. In human AR, a small case-control study of stool from 13 adults with AR and 10 healthy individuals reported that the AR cohort had greater *Bacteroidetes* abundance and lower *Firmicutes* abundance (83). Zhou reported consistent findings (84). And Liu also compared 93 patients with AR to 72 healthy controls and found reduced gut microbiota diversity, increased *Bacteroidetes*, decreased *Actinobacteria* and *Proteobacteria*, and enrichment of *Escherichia-Shigella*, *Prevotella*, and *Parabacterium* (85). In 1,092 adults, Yamaguchi examined blood and fecal samples and discovered that higher *Bacteroidetes* abundance was associated with allergen sensitization, while lower *Firmicutes* abundance was associated with symptom manifestation (86). Watts also observed a decrease in *Oxalobacter* abundance in the AR group, which is important for oxalate metabolism and preventing oxalate-related disorders such as calcium oxalate kidney stones (83). Some of these human studies, particularly the Watts case–control cohort, are limited by modest sample sizes, cross-sectional design, dietary and medication confounding, and fecal rather than mucosal sampling.

In contrast, in an OVA-induced AR mouse model, the AR group exhibited increased proportions of *Firmicutes* and *Proteobacteria*, lower *Bacteroidetes* abundance, and lower fecal SCFA concentrations (87, 88). This apparent human-animal discrepancy is not necessarily contradictory once its sources are considered: host species and gastrointestinal physiology differ; standardized chow, specific-pathogen-free housing, and genetic homogeneity in model mice contrast with the heterogeneous diets, environments, and medication exposure of patients; 16S rRNA pipelines and reference databases differ between studies; fecal pellets are not equivalent to human stool; and OVA models reproduce an induced, acute Th2-skewed phenotype rather than chronic, naturally occurring human AR. Cross-species comparisons should therefore be made at the level of functional pathways rather than individual taxa (**Figure 4**).

**Figure 4:**
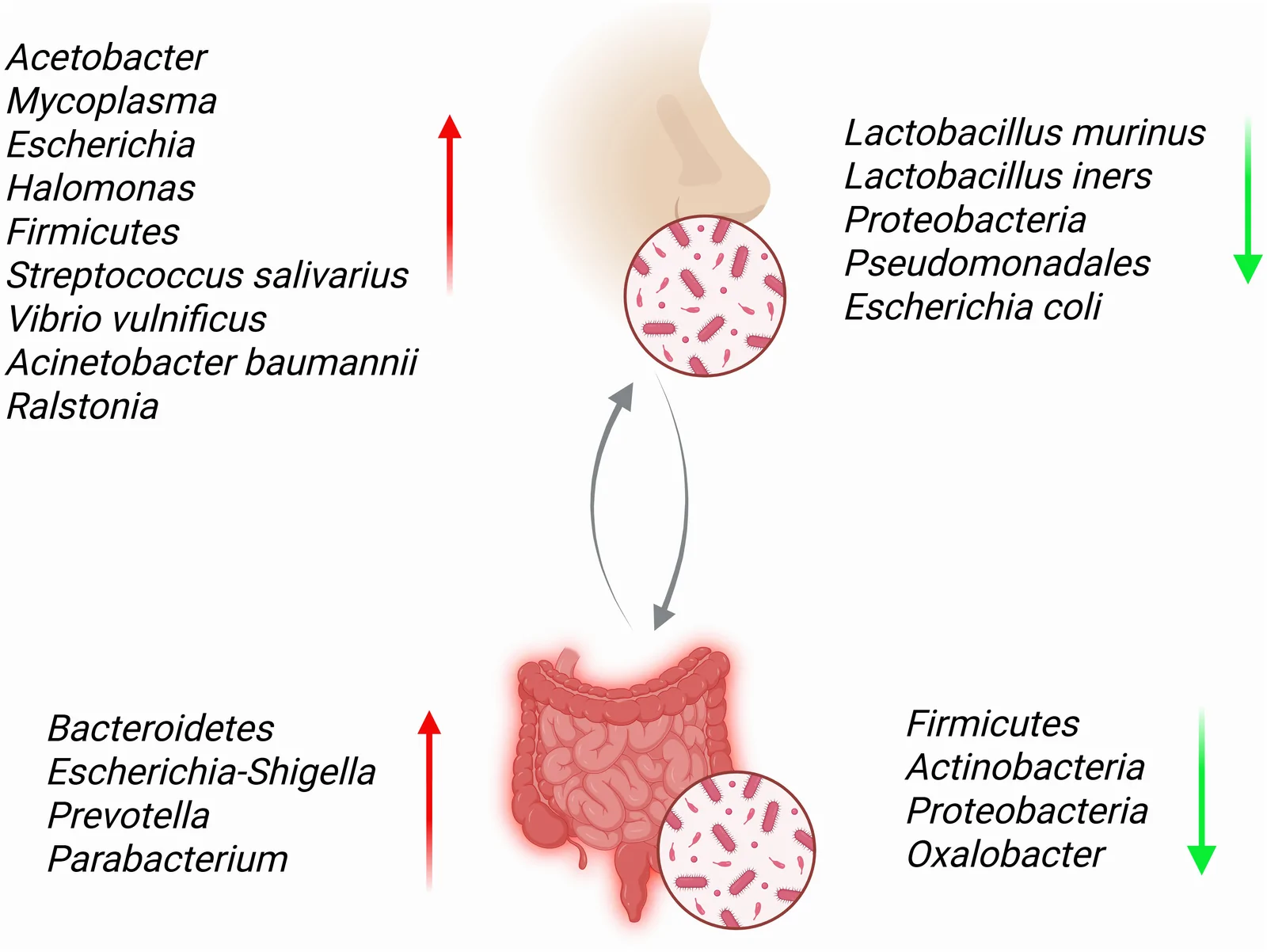
Alterations in nasal and gut bacterial communities along the gut-nasal axis. Red upward arrows indicate bacterial taxa reported at increased relative abundance, green downward arrows indicate taxa reported at reduced relative abundance, and the bidirectional arrow represents reciprocal crosstalk between the nasal and gut microbial compartments. Because the direction of change differs among studies and between human cohorts and mouse models, arrows depict reported trends rather than a validated, reproducible signature. Abbreviations: SCFA, short-chain fatty acids; F/B, Firmicutes-to-Bacteroidetes ratio.

## 5 Microbiota-targeted therapeutic strategies and their mechanisms

Given the emerging role of microbial dysbiosis in AR, targeting microbiota has emerged as a promising therapeutic approach. Probiotics, prebiotics, synbiotics, bacterial lysates (BLs), and fecal microbiota transplantation (FMT) are all interventions designed to restore microbial homeostasis and modulate the host immune response. Below, we discuss the current evidence and proposed mechanisms of action for the primary microbiota-targeting strategies.

### 5.1 Probiotics

Probiotics, defined as live microorganisms that provide a health benefit to the host when administered in adequate quantities, are the most extensively studied microbiota-targeting therapy for AR. Clinical trials on various strains, primarily *Lactobacillus* and *Bifidobacterium* species, have yielded mixed but generally positive results in terms of nasal symptom scores and quality of life (89–92). Importantly, benefit from one strain cannot be generalized to probiotics as a class, and individual randomized trials of several strains—including an *L. paracasei* LP-33 trial and some *Lactobacillus rhamnosus* GG trials—reported no nasal-symptom benefit, whereas other trials of related strains did, reinforcing that efficacy cannot be extrapolated across strains; apparent efficacy also depends on dose (colony-forming units, CFU), treatment duration, age, seasonal versus perennial AR, concomitant pharmacotherapy, and the chosen outcome instrument.

#### 5.1.1 Human clinical evidence

Human RCTss in AR differ markedly in design, strain, dose, duration, population, and outcome instruments, so findings cannot be extrapolated across products; doses, duration, populations, and principal outcomes are compiled in **Table 3**. The NVP-1703 mixture (*Bifidobacterium longum* IM55 plus *Lactobacillus plantarum* IM76) reduced total nasal symptom score (TNSS) and maintained IL-10/IL-4 and IL-10/IL-13 ratios (93). Among the better-controlled trials, a synbiotic regimen significantly improved TNSS, whereas visual analog scale (VAS) and Rhinoconjunctivitis Quality of Life Questionnaire (RQLQ) scores improved only as non-significant trends (94). Fermented milk containing *Lactobacillus helveticus* SBT2171 lowered nasal fluid and blood eosinophil counts and reduced nasal congestion in perennial AR (95). In children, *Bifidobacterium animalis subsp. lactis* BB-12 plus *Enterococcus faecium* L3 reduced AR symptoms and the use of rescue antihistamines and intranasal corticosteroids (90). Pooled meta-analyses report only modest average effects with high statistical heterogeneity: nasal symptom scores improved significantly whereas global and ocular symptom scores did not, and benefit was not reproduced for every strain or outcome instrument (91, 92).

**Table 3:** Human randomized controlled trials of probiotics/synbiotics in AR.

Preparation (strain)	Dose	Duration	Population & design	Principal outcome & result	Adverse events	Ref.
probiotic mixture (B*. animalis* subsp. *lactis* BB12 DSM 15954 + *Enterococcus faecium* L3 LMG P-27496)	2×10^9^ CFU of each strain daily	3 months (pre-season prophylaxis)	250 children aged 6-17; randomized, double-blind, placebo-controlled (add-on to standard therapy)	AR signs/symptoms ↓, rescue antihistamine and intranasal corticosteroid use ↓	Well tolerated; no serious AEs	(90)
NVP-1703 (*B. longum* IM55 + *L. plantarum* IM76)	1×10^10^ CFU/sachet daily	4 weeks	95 adults with perennial AR; randomized, double-blind, placebo-controlled	TNSS ↓, maintained IL-10 and IL-10/IL-4, IL-10/IL-13 ratios	No product-related AEs; none serious	(93)
Probiotic + prebiotic synbiotic	Twice daily (formulated sachets)	90 days + 30-day follow-up	Adults with seasonal AR; randomized, double-blind, placebo-controlled; loratadine rescue	TNSS significant (VAS/RQLQ: non-significant trends) ↓, acetate, TNF-α/IFN-γ ↑, IL-17 ↓	No serious AEs; mild, mainly gastrointestinal	(94)
*Lactobacillus helveticus* SBT2171 (fermented milk)	Daily fermented-milk formulation	16 weeks	Japanese adults with mild-moderate perennial AR; randomized, double-blind, placebo-controlled	Nasal fluid and blood eosinophils ↓, nasal congestion ↓	No product-related AEs; none serious	(95)

#### 5.1.2 Preclinical evidence

Murine OVA- or pollen-induced AR models permit mechanistic control but cannot by themselves establish clinical efficacy. In these models, *B. longum* IM55 plus *L. plantarum* IM76 and several other *Lactobacillus* and *Bifidobacterium strains*, along with intranasally instilled probiotic extracts, variably reduced symptoms, allergen-sIgE, and Th2 cytokines, with reported Th2/Treg or Th1/Th2 rebalancing (96–100) (**Table 4**). Strain, human-equivalent dose, and route (oral versus nasal) differ across studies, and positive outcomes in genetically homogeneous mice should be treated as hypothesis-generating.

**Table 4:** Preclinical probiotic studies in AR models.

Strain/preparation	AR model	Route	Main outcome	Proposed mechanism	Ref.
*B. longum* IM55 + *L. plantarum* IM76	Female BALB/c mice model of OVA AR	Oral	Alleviated nasal symptoms and inflammation.	Restored Th2/Treg imbalance and gut microbiota disturbance.	(96)
*L. plantarum* CJLP133, CJLP243	Mice model of birch-pollen AR	Oral	Alleviated symptoms and reduced IgE.	Regulated Th1/Th2 balance.	(97)
*Lactiplantibacillus plantarum* NR16	Murine AR model	Oral	Ameliorated symptoms and IgE.	Suppressed Th2 cytokine production.	(98)
*Bifidobacterium breve*	Female BALB/c mice model of OVA AR	Oral	Improved allergic symptoms and reduced sIgE.	Shifted Th2 toward Th1 profile.	(99)
*Clostridium butyricum* extracts	Male BALB/c mice model of OVA AR	Nasal instillation	Inhibited allergic nasal response.	Improved Th1/Th2 cytokine balance of nasal mucosa.	(100)

#### 5.1.3 Mechanistic evidence

Multiple non-exclusive mechanisms have been proposed across different strains. DCs conditioning, more importantly than a simple Th1 shift, triggers Tregs that produce IL-10 and TGF-β. Some strains also raise IL-12 and IFN-γ levels (93). Reinforcement of intestinal tight junction integrity restricts the translocation of luminal antigens. SCFA signaling, including butyrate propionate and acetate, drives Treg differentiation and inhibits histone deacetylases. Interventions modulate the gut nasal/lung axis through circulating metabolites and immune cells. The relative contributions of these mechanisms vary by strain. Live probiotics, inactivated preparations, and purified metabolites represent separate intervention categories. The early concept of Th1/Th2 rebalancing is not universally applicable. Many effective strains exert effects primarily through tolerogenic DCs and Tregs (96). They may suppress Th17 responses or function through metabolites. Some strains change neither IFN-γ nor Th1 markers. Elevated IL-12 and IFN-γ have been observed only for selected strains. This finding cannot be generalized.

Probiotics can reinforce the intestinal epithelial barrier through the upregulation of tight junction proteins. Direct effects on the nasal epithelium remain poorly defined. Reductions in systemic inflammation driven by gut modulation may indirectly preserve nasal barrier integrity. Certain probiotic strains and commensal bacteria generate SCFA. Butyrate supports extrathymic Treg differentiation and inhibits class I/II histone deacetylases (94). SCFA synthesis depends on substrate availability and strain characteristics. This outcome can also be attained using prebiotic fibers without live probiotic administration.

### 5.2 BLs

BLs are extracts of chemically or mechanically inactivated bacteria, typically administered orally or sublingually, that mimic the immunomodulatory effects of broad microbial exposure without the risk of infection. This approach is aligned with the hygiene hypothesis. BLs differ fundamentally from live probiotics, which contain non-replicating organisms, and from purified metabolites, which contain mixed pathogen-associated molecular patterns. They activate PRR/TLR pathways on APCs, promote tolerogenic DCs and IL-10-mediated Treg responses, increase mucosal secretory IgA, and shift immune responses away from exclusive Th2 polarization instead of non-specifically boosting Th1 immunity (**Table 5**). The available evidence for BLs is promising yet limited in quantity. Researchers must clearly define source mixture, dosing cycle, administration route, and patient phenotype in all relevant studies.

**Table 5:** BLs in AR.

Preparation	Regimen (dose/duration)	Population & design	Key result	Adverse events	Ref.
OM-85 BV	Animal experiment	Mice infected with influenza and Salmonella	Protected against infection (infection endpoint, not AR)	Not applicable.	(118)
OM-85 BV	7 mg capsule, 10 days/month for 3 months	84 patients (29 with AR) with AR/asthma/COPD aged 16-65; open-label sequential study	Reduced respiratory infections	Mild and tolerable, and no serious AE.	(119)
Broncho-Vaxom	7 mg/day for 10 days, 20-day rest, 3 cycles	60 adults with persistent AR; randomized placebo-controlled trial	symptom scores ↓, IFN-γ in nasal lavage ↑, IL-4/IL-13 and eosinophils ↓	No severe adverse events, but 3 cases of mild self-limiting abdominal pain.	(120)
Polyvalent mechanical BLs	1 tablet/day for 10 days, 20-day break, 3 cycles	70 children aged 5–17 with grass-pollen AR; randomized placebo-controlled trial	TNSS and nasal VAS ↓, peak nasal inspiratory flow ↑	Well tolerated and with no serious AE.	(121)

### 5.3 FMT

FMT is the most comprehensive gut microbiota-targeted strategy, involving the transfer of the entire microbial community from a healthy donor to restore gut microbial ecology and recalibrate systemic immunity. Its evidence base in AR, however, is essentially absent: no published RCTs has evaluated FMT for AR (**Table 6**). These data support biological plausibility for the gut-nasal/lung axis, encompassing bacterial metabolites, HDAC inhibitors such as sodium butyrate, and microbiome-derived vesicles, yet they do not demonstrate clinical efficacy of FMT for AR (101–103, 123–126). FMT should not be regarded as having equivalent evidence to probiotics or BLs.

**Table 6:** FMT and gut microbiota-targeted approaches.

Intervention/focus	Evidence context for AR	Population/model	Key Findings	Proposed mechanisms	Ref.
FMT	Chronic urticaria, abdominal pain, and food allergy	Patient case series	Improvement in urticaria and abdominal pain.	Restoring healthy gut microbiota to modulate systemic immunity.	(101, 122)
Sodium butyrate	AR model	Mouse model of AR	Therapeutic effect on AR.	Histone deacetylase inhibition.	(102)
*E. coli* O83 extracellular vesicles	Allergic airway inflammation	Mouse model	Prevented allergy development.	Activated innate immunity.	(103)
Gut microbiota & metabolites	Atopy cohorts, not AR-specific	Human cohorts and mouse models	High early-life butyrate/propionate protective; 12,13-diHOME impedes tolerance.	Gut bacteria produce metabolites that influence immune tolerance.	(123, 124)
Bacterial histamine secretion	Lung allergy model	Mouse model	Gut-derived histamine aggravated lung allergic responses.	Bacterial secretion of histamine within the gut influences immune responses within the lung.	(125)
Gut microbiome composition	Observational AR association	Childhood AR cohort	Gut composition associated with AR and HDM IgE.	Gut microbiota dysbiosis influences immune development and allergic sensitization.	(126)

The safety and regulatory burden of FMT also surpasses that of orally administered probiotics or BLs. Procedure-related adverse events include aspiration during upper-gastrointestinal delivery, perforation or bleeding with colonoscopic delivery, and sedation-related reactions. Microbiological risks include transmission of multidrug-resistant organisms and other pathogens. Rigorous donor screening, screened or quarantined stool banking, investigational oversight, informed consent that explicitly discloses the experimental status of FMT in AR, and enhanced protection of vulnerable patients are prerequisites for any future trial.

In conclusion, microbiota-directed interventions operate via partially overlapping pathways, metabolite signaling, epithelial barrier support, and reorientation of innate and adaptive immunity, yet their levels of evidence differ substantially. Specific probiotic strains and BLs are backed by small RCTs that demonstrate modest symptomatic benefits. Prebiotics, synbiotics, and microbial metabolites are at earlier developmental stages. FMT remains experimental for AR. Future studies should standardize strain-level reporting, dose and treatment duration, clinically meaningful endpoints, and systematic adverse-event recording to refine treatment strategies for AR.

## 6 Controversies and future directions

The relationship between microbiota and AR has sparked debates about causation and correlation. Most human data are cross-sectional, so dysbiosis could precede and contribute to AR, result from allergic inflammation or medication, or share upstream environmental causes. A Mendelian randomization analysis reported a genetically predicted causal association between gut microbiota and AR, specifically *Ruminococcus gauvreauii*, and an increased risk of AR, thereby supporting this causal relationship (104). However, Mendelian randomization rests on a set of assumptions that are challenging to satisfy in this research setting: horizontal pleiotropy, weak instrument bias arising because host genotypes explain only a small fraction of inter-sample taxonomic variance, limited statistical power, and population stratification. Furthermore, microbiome composition and function are continuously shaped by host diet and environmental factors after an individual’s genotype is established. Accordingly, current findings should be interpreted as hypothesis-supporting rather than definitive and require independent replication in separate cohorts. Longitudinal birth cohorts, gnotobiotic animal models, and microbiota transfer experiments have yielded complementary yet inconclusive causal evidence to date; resolving these methodological uncertainties is therefore critical to developing effective clinical diagnostic and therapeutic strategies targeting the microbiome.

Reliable, comparable microbiome evidence hinges on standardized methodology, and multiple technical limitations warrant systematic consideration. Marker-gene 16S rRNA sequencing resolves taxa mainly to the genus level and exhibits region-dependent performance, for example, V1-V2 versus V3-V4, whereas shotgun metagenomics provides species- and gene-level resolution at higher cost. Microbiome data are compositional. Relative abundances sum to a constant, so an apparent rise in one taxon may merely reflect a decline in another, necessitating compositional data analysis approaches. Batch effects originating from DNA extraction kits, sample storage, and library preparation can outweigh biological signals. The DNA extraction process heavily influences variability in metagenomic analysis of fecal microbiome composition, which has a significant impact on gut microbiota diversity. Using different DNA extraction kits and sample storage techniques leads to inconsistent findings, emphasizing the need for standardized operational protocols (105). In South Korea, the Clinical-Based Human Microbiome Research and Development Project has established standardized protocols for clinical metadata collection, specimen management, DNA extraction, sequencing, and quality control implementation (106). Nasal samples are low-biomass and particularly susceptible to reagent and environmental contamination, which must be quantified using extraction blanks and negative controls. Sequencing depth, rarefaction procedures, and taxonomy-resolution choices bias diversity estimates. Relative abundance cannot capture absolute bacterial load, supporting the use of spike-in quantitative methods. Bacterial profiling in isolation ignores fungal and viral compartments, while integrated metabolomics links microbial community structure to functional outputs. Cross-sectional designs cannot establish temporal ordering. Repeated longitudinal sampling, ideally collected prior to treatment and across seasonal time points, is necessary. Diet-microbiome research further requires validated dietary assessment tools and sufficient statistical power. Standardization across all these dimensions is a prerequisite for identifying replicable AR-associated microbiome biomarkers.

Probiotics, prebiotics, BLs, and microbial metabolites may complement AR management, but the optimal strain, dosage, route, timing, and treatment duration remain to be defined. FMT in particular must be approached with caution, with rigorous informed consent and protection of vulnerable patients rather than selection driven by patient desperation (107). Additionally, traditional Chinese medicine such as Xiaoqinglong decoction has also been found to reduce nasal inflammation in AR mice by regulating gut microbiota imbalance, suggesting that microbiota modulation may be a shared downstream pathway of disparate interventions (108).

Nanoparticles are under investigation as potential adjuvants for allergen immunotherapy to enhance safety, shorten treatment duration, and increase local drug concentrations (109). Analogous encapsulation and extracellular vesicle platforms may preserve live biotherapeutics and microbial metabolites during gastrointestinal transit. These approaches are still at the preclinical stage, yet they bridge microbiome science with delivery-system engineering and underscore the wide range of microbiota-targeted strategies for AR.

## 7 Conclusions

In conclusion, the microbial community maintains a complex, bidirectional relationship with AR pathogenesis, and microbiota-directed therapies hold conditional rather than firmly established therapeutic promise.

The strongest conclusions supported by current evidence span three core domains. Early-life microbial exposure and nasal or gut dysbiosis are associated with AR risk, yet human evidence remains largely observational and causality continues to be debated. Mechanistically, pathogenic pathways converge on epithelial barriers, alarmins, ILC2s, DCs subsets, Tregs, Th17 cells, and microbial metabolites, rather than being driven by a Th1/Th2 shift alone. In terms of interventional evidence, selected probiotic strains and BLs have modest, strain-specific RCTs data supporting symptom relief, while FMT remains an experimental strategy for AR.

The field’s most critical unanswered questions include whether a reproducible, clinically validated nasal or gut microbial signature of AR exists; which microbial functions, rather than individual taxa, exert causal effects; which combinations of strain, dose, treatment duration, and patient age yield clinically meaningful benefits; and how disease activity and medications in turn reshape the microbiome.

A focused research agenda should prioritize longitudinal, adequately powered, confounder-adjusted human studies incorporating absolute quantitation and multi-omics approaches; standardized sampling and analysis protocols; preregistered, strain-specific RCTs measuring patient-important outcomes and reporting adverse events; and mechanistic investigations using gnotobiotic models and metabolite experiments. Pursued in this rigorous framework, nasal and intestinal microbiota research could progress from observational associations toward genuinely preventive or therapeutic applications in AR.
